# Carbon footprint of Nepalese healthcare system: A study of Dhulikhel Hospital

**DOI:** 10.12688/f1000research.139552.1

**Published:** 2023-10-18

**Authors:** Bikash Adhikari, Ambika Dangal, Sushila Pandey, Bijay Thapa, Ashim Joshi, Bivek Baral

**Affiliations:** 1 Department of Environmental Science and Engineering, Kathmandu University, Dhulikhel, Bagmati, 45200, Nepal; 2 Department of Mechanical Engineering, Kathmandu University, Dhulikhel, Bagmati, 45200, Nepal

**Keywords:** greenhouse gas, life cycle assessment, health care sectors

## Abstract

**Background:** Though direct greenhouse gas emissions cannot be observed in health care sectors, there can exist indirect emissions contributing to global climate change. This study addresses the concept of the carbon footprint and its significance in understanding the environmental impact of human activities, with a specific emphasis on the healthcare sector through gate-to-gate (GtoG) life cycle assessment. Transportation, energy consumption, and solid waste generated by hospitals are the primary sources of carbon emissions. **Methods:** Different standards, guidelines and parameters were used to estimate emissions from both the primary and secondary data. All steps and sub-steps involved in GtoG were accessed and analyzed within the standard ISO 14040:44 guideline. An extensive review of existing literature was carried out for the evaluation and verification of secondary data. **Results:** The total carbon footprint of generators, electricity consumption, transportation activities, LPG cylinders, PV systems was found to be 58,780 kg-CO2-eq/yr, 519,794 kg-CO2-eq/yr, 272,375 kg-CO2-eq/yr, 44,494 kg-CO2-eq/yr, 35,283 kg-CO2-eq/yr respectively and the emissions from non-biodegradable solid waste was found to be 489,835 kg-CO2/yr. Local air pollutants such as PM _10_, CO, SO _2_, NO _X_, and VOCs generated by generators and transportation were also estimated. The CH _4_ emissions from liquid waste were 1177.344 kg CH _4_/BOD yr, and those from biodegradables were 3821.6954 kg CH4/yr. **Conclusions:** Healthcare professionals and policymakers can take action to reduce the sector's carbon footprint by implementing best practices and encouraging sustainable behavior. This study can be taken as foundation for further exploration of indirect emissions from healthcare sectors not only in Nepal but also in south Asian scenario.

## Introduction

Direct emissions in the healthcare industry are relatively low as compared to other sectors. ^1^ However, a sizable portion of the country's CO _2_ footprint can be attributed to emissions along the supply chain brought on by the healthcare sector's purchases of goods and services. ^1^^,^^2^ In terms of energy consumption, hospitals and other healthcare facilities are major users of energy for heating, cooling, lighting, and the operation of medical equipment. The provision of healthcare and the acquisition of goods, energy use, transportation, services, and technological advancements from a carbon-intensive supply chain all contribute to the direct and indirect emission of greenhouse gasses (GHGs) in every country. ^3^ Although health care services are essential for maintaining and enhancing human wellbeing, they also have an environmental impact that adds to the environmental risks to human health. Recently, concerns regarding how to make medical practices more sustainable due to the carbon footprints linked with healthcare have emerged. ^4^^,^^5^ Healthcare produces a variety of waste streams, each with a distinctive method of disposal and thus a different carbon impact. The material composition and method of disposal, as well as the available options, determine the carbon footprint emerging from healthcare facilities. In order to access such emissions, life cycle assessment (LCA) is a suitable form. ^6^^–^^8^

Due to the increased GHG emissions brought on by human activity, climate change is a global phenomenon that affects all sectors. ^9^ The carbon footprint of health care has been calculated previously in some of the developed countries, including the UK, the USA, Australia, Canada, China, Japan. International comparisons and the studies estimated the health care GHG footprint as percentage of the national GHG footprint to be 8–10% in the US, 3% in England, 7% in Australia, and 5% in Canada. ^3^ The entire CO _2_ eq emissions in Australia in 2014–15 was 494,930 kilotonnes, and the health care sector accounted for 35,772 (7%) of those emissions. According to total CO _2_ eq emissions, the five most significant areas of the healthcare industry were: other medications (3347 kilotonnes [9%]), benefit-paid drugs (3257 kilotonnes [9%]), public hospitals (12,295 [34%] of 35,772 kilo tonnes CO _2_ eq), and capital costs for buildings (2776 kilotonnes [8%]). ^2^ There were significant standby energy emissions from scanners. ^10^ The majority of the carbon footprints left by MRIs and CTs are driven by their heavy reliance on electricity, especially standby power. ^10^ For coagulation profile testing, CO _2_-eq emissions were 82 g/test (95% CI, 73–91 g/test), and for whole blood examination tests, they were 116 g/test (95% CI, 101–135 g/test). CO _2_ -eq emissions for biochemical tests were 49 g/test (95% CI, 45–53 g/test) for arterial blood gas assessment, 99 g/test (95% CI, 84–113 g/test) for urea and electrolyte assessment, and 0.5 g/test CO _2_ eq (95% CI, 0.4–0.6 g/test) for C-reactive protein (low because it is frequently ordered with urea and electrolyte assessment). Most CO _2_-eq emissions (ranging from 60% for whole blood examination to 95% for coagulation profile) were related to sample collection; emissions related to laboratory reagents and electricity use were substantially smaller. ^10^ Japanese healthcare services had an overall carbon footprint of 62.5 × 106 metric tons of CO _2_ equivalent (Mt CO2 -eq) in 2011, which is 4.6% of all domestic GHG emissions. ^11^ The entire carbon footprint increased by nearly 15% in just four years, reaching 72.0 metric tonnes CO _2_-eq in 2015. ^11^ China invested CNY 2539 billion on healthcare in 2012, which generated 315 (68% CI 267–363) megatonnes of CO _2_-eq emissions. A total of 27% (68% CI 23–31) of China's GHG emissions were attributable to the health sector. ^12^ Public hospitals (148 megatonnes [47%]), non-hospital purchased medications (56 megatonnes [18%]), and building (46 megatonnes [15%]) were the main contributors to GHG emissions in the healthcare sector. ^12^ Only 16% of the carbon footprint in medical facilities was produced by energy use for buildings and transportation; the other 84% was caused by the goods and services that have been purchased. ^12^ The carbon footprint of waste streams in a UK hospital was assessed in 2022 using activity data from three hospitals in one UK National Health Service organization, in accordance with the Greenhouse Gas Accounting Sector Guidance for Pharmaceutical Products and Medical Devices. In England, the National Health Service (NHS) produces 538,600 tonnes of waste per year. According to the study, recycling has the lowest carbon footprint per tonne of hospital waste (21–65 kg CO _2_-eq), followed by low temperature incineration with energy from waste (172–249 kg CO _2_-eq). ^13^

The primary objective of this study is to estimate the carbon print of hospitals of Nepal, whereas it also aims to recommend majors that can be used to reduce the emissions from such public sectors. It provides a complete and clearer picture of carbon footprint of south Asian scenario and can be set as a foundation for further case studies.

## Methods

### Goal and scope

The field of study for this study is Dhulikhel Hospital. Dhulikhel Hospital, a Kathmandu University hospital has 18 health centers with 475 medical beds. Dhulikhel hospital is one of the largest hospitals of Nepal. The functional unit considered is kg CO _2_-eq per year, with the consideration of transportation mediums life cycle of 20,000 km per year. A gate-to-gate analysis has been employed considering the simplicity of analysis, mainly due to the involvement of considerable numbers of sectors to be assessed.

### Material and energy flow

The hospital has the capability to utilize up to 1430 KVA of electricity, although the actual demand stands at 500 KVA. To meet its power requirements, the hospital employs four generators, with capacities of 250 KVA, 160 KVA, and two generators of 100 KVA each.

In terms of transportation, Dhulikhel Hospital maintains a fleet of 16 vehicles specifically dedicated to hospital services, comprising a mix of diesel and petrol vehicles. Additionally, the hospital possesses 52 private cars, out of which 44 operate on petrol, 5 on diesel, and 3 are electric vehicles.

For its energy needs, the hospital relies on four solar panels, collectively possessing a capacity of 285 kWh.

Regarding waste management, Dhulikhel Hospital generates a daily average of 503.51 kg of non-risk waste, 258.21 kg of risk waste, and 167.78 kg of biodegradable waste. The generated waste is collected, segregated, and subsequently transported to appropriate disposal sites and recycling centers.

**Figure 1.**
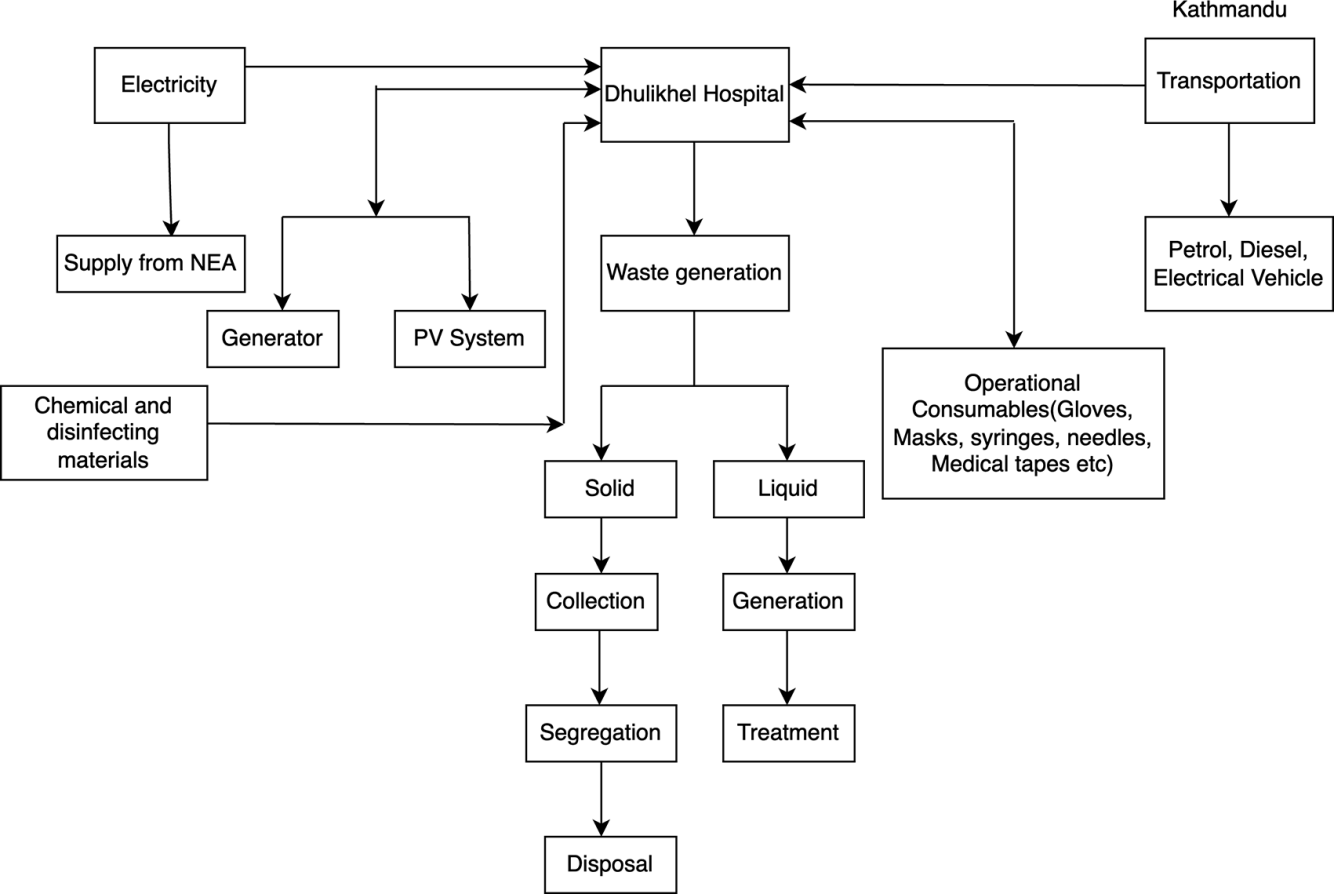
Energy and material flow in Dhulikhel Hospital system.

To address waste treatment requirements, the hospital has implemented a medium-sized system, incorporating three phases of treatment. This system comprises an Anaerobic Baffled Reactor, a Horizontal Wetland, and a Vertical Wetland, with two systems operating simultaneously. The combined treatment capacity of these systems is 100 m ^3^/day. See Figure 1 for the energy and material flow of the system.

### Primary and secondary data collection

ISO 14040:44 standard was applied during the primary data collection. Intergovernmental Panel on Climate Change (IPCC) ^14^ guideline and U.S. Environmental Protection Agency (EPA) ^15^ guideline have been used to calculate vehicle emissions along with previous data. National electricity carbon footprint of Nepal has been calculated by modifying the data available from Joshi *et al.* ^16^ In the case of life cycle emission of solar systems, total capacity of the system was multiplied by the life cycle emission provided by Mehedi *et al.* ^17^ Furthermore, waste reduction method was applied to conduct waste audit to categorize biodegradable and non-biodegradable components to estimate their respective carbon footprints. An average of 10 solid waste samples from each month (December 2022–March 2023) was taken for consideration. Nepal Electricity Authority (NEA) installed smart meter at the hospital was observed to estimate hospital energy consumption, whereas average electricity outage per day was calculated according to the data provided from the authority itself. Normalization of data was done according to the functional unit. Onsite visits to the landfill were conducted to perform grab sampling of solid waste and of liquid waste. The calculations of emissions from each source are explained in respective sections. All data used in the study are available in the data file. ^27^

As for secondary data, it involved extensive analysis of existing data from sources such as government reports, academic studies, and industry publications. These includes Dhulikhel Hospital and Nepal Electricity Authority and others as referenced studies. Information includes data on energy consumption, operational consumables and liquid waste generation as well as data on the environmental impact of the production and disposal of medical equipment and supplies. Additionally, data from medical research studies were analyzed to assess the environmental impact of certain treatments and procedures. Secondary data were at least validated, by comparing and contrasting with other two sources of last five years. Greet Model ^18^ provided by Argonne National Laboratory was also used for the calculation of vehicle emission updating the electricity carbon footprint of Nepal.

### Estimating generator emission

Dhulikhel Hospital has four generators, 250 KVA, 160 KVA and two generators of 100 KVA capacity.

The equation used for the estimation of the emissions:

$$E_{i}=P\times O_{p}\mathrm{Hrs}\times EF_{i}\times \left[\frac{100-ER_{i}}{100}\right]\times \mathrm{LF}$$

where,

E _i_ = Total emission of substance i from a stationary combustion engine for the reporting year (kg/yr)

P = Engine power capacity rating (kW)

O _p_Hrs = Operating hours of engine during the reporting year (h/yr)

EF _i_ = Emission factor of substance i (kg/kWh)

ER = Emission reduction efficiency for substance i (%)

I = Substance i (-)

LF = Load Factor 75%

Since the pollution control technology employed in the diesel generator sets is not clearly known, the emission reduction efficiency is zero and thus the emissions results are 'uncontrolled' emission values. ^19^ Table 1 provides the emission factors for the estimation of total carbon dioxide equivalent emission.

**Table 1.** Emission factors for emission estimation (Generator). ^15^

	For newer engines >15 years old		For old engines, >15 years
	Emission Factor (g/kWh)		
Substance	< 447 Kw	> 447 kW	Old engine of all size
Carbon monoxide, CO	4.06	3.2	
Oxides of nitrogen, NO _X_	18.8	14	
Particulate matter (PM10)	1.34	0.33	4.5
Sulfur dioxide, SO _2_	0.18	0.18	
Total Volatile organic compounds, VOC	1.5	0.43	
Carbon dioxide (CO _2_)	704	703	

### Estimating emission due to electricity consumption

Hospitals are energy-intensive buildings that require a constant and reliable supply of electricity to operate critical medical equipment. In Nepal, most hospitals rely on the national grid for their energy needs. Dhulikhel Hospital can use electricity up to 1430 KVA but the actual demand is 500 KVA. Table 2 shows the estimation of carbon intensity of Nepali electricity.

$$\text{Carbon footprint emission},\left(\mathrm{kg}\;CO_{2}-\mathrm{eq}/\mathrm{yr}\right)=\left(\text{Emission factors},\left(g\;CO_{2}-\mathrm{eq}/\mathrm{kWh}\right)\times \text{consumed unit},\left(\mathrm{kWh}/\mathrm{yr}\right)\right)/1000$$

**Table 2.** National Electricity Carbon Footprint of Nepal.

S. N	Process	Emission factor	Unit	Remarks	References
1	NEA Electricity	240.26	g-CO _2_-eq/kWh	Weighted average of Nepal Produces Electricity and Indian Import with T&D loss of 15%	^16^

### Estimating emission due to different modes of transportation

Dhulikhel hospital has 16 different diesel and petrol vehicles for hospital services and 52 private cars in which 44 are found to be petrol cars, five are diesel and three are electric vehicles, found by random sampling. Table 3 and 4 show the emissions generated from IC vehicles and carbon footprint of electric vehicles respectively.

$$\text{Total emission for local pollutants}\left(\mathrm{kg}/\mathrm{yr}\right)=(\text{Total traveled distance annually}\;\left(\mathrm{km}/\mathrm{yr}\right)\;\times \;\text{emission factors}\;\left(g\;CO_{2}-\mathrm{eq}/\mathrm{km}\right))/1000$$

$$\text{Total carbon emission}\;\left(\mathrm{kg}\;CO_{2}-\mathrm{eq}/\mathrm{yr}\right)=\text{Total distance traveled annually},\left(\mathrm{km}/\mathrm{yr}\right)\times \text{Fuel efficiency},\left(\mathrm{km}/L\right)\times \text{emission factor},\left(\mathrm{kg}\;CO_{2}-\mathrm{eq}\right)$$

**Table 3.** Emissions for IC vehicles. ^20^

Vehicle type	PM 2.5 (g/km)	NO _x_ (g/km)	CO _2_ emission (kg-CO2-eq)	CO (g/km)	VOC (g/km)	Fuel efficiency (km/L)
Petrol vehicles	0.05	0.144271287	2.31	8.369	0.64	15.75
Diesel vehicles	0.162	0.657	2.68	2.244	0.547	12
Bus	2.672	21.38	2.68	11.92	3.311	4.25

**Table 4.** Emission Factor for Electric vehicle.

Working days (day)	Average distance traveled per day, (km/day)	Emission Factor, (g-CO2-eq/km)	Reference
265	70	109	^16^

For electric vehicles,

$$\text{Total emission}\;\left(\mathrm{kg}\;{\mathrm{CO}}_{2}-\mathrm{eq}/\mathrm{yr}\right)=\left(\text{Working days}\left(\mathrm{day}\right)\times \text{Average distance traveled}\;\mathrm{per}\;\mathrm{day}\left(\mathrm{km}/\mathrm{day}\right)\times \;\text{Emission factor}\left(g\;{\mathrm{CO}}_{2}-\mathrm{eq}/\mathrm{km}\right)\right)/1000$$

### Estimating emissions from LPG cylinder

Dhulikhel hospital uses 1020 cylinders of gas weighing 15.6 kg per cylinder in a year.

$$\text{Total emission}\;\left(\mathrm{kg}\;CO_{2}-\mathrm{eq}/\mathrm{yr}\right)=\mathrm{No}.\text{of cylinders used}\;\mathrm{per}\;\text{year}\times \text{Average weight of}\;a\;\text{cylinder}\left(\mathrm{kg}\right)$$

$$\times \;\text{Conversion Factor}\left(L/\mathrm{kg}\right)\times \text{Emission Factor}\;\left(\mathrm{kg}-CO_{2}-\mathrm{eq}/L\right)$$

For the conversion factor, the density of LPG at standard conditions, which is approximately 0.54 kg/L. This means that 1 liter of LPG at standard conditions ( *i.e.*, a temperature of 15 °C and a pressure of 1 atm) has a weight of 0.54 kg. Table 5 shows the emission generated from LPG.

**Table 5.** Emission factor for LPG.

No of cylinders used, per month	No. of cylinders used, per year	Average weight of a cylinder (kg)	Conversion Factor (L/kg)	Emission Factor, (kg-CO2-eq/L)	Reference
85	1020	15.6	0.54	1.51	^14^

### Estimating emissions from PV system

Dhulikhel hospital has four solar panels having 285 kWh capacity in total. Table 6 shows the carbon footprint of PV system.

$$\text{Total emission},\left(\mathrm{kg}\;CO_{2}-\mathrm{eq}\right)=(\text{capacity of}\;\mathrm{PV}\;\text{system},\left(\mathrm{kWh}\right)\times \text{Emission Factor},(g\;CO_{2}-\mathrm{eq}$$

/kWh))/100 0

**Table 6.** Emission factor for PV system.

PV system capacity (kWh)	Emission Factor (g-CO2-eq/kWh)	Reference
285	123.8	^17^

### Estimating emissions from solid waste generation

Dhulikhel hospital generates 503.51 kg of non-risk waste per day and generates 258.21 kg of risk waste per day. Table 7 provides the emissions factors for the respective types of waste.

$$\text{Total carbon emission}\;\left(\mathrm{kg}\;CO_{2}/\mathrm{day}\right)=\text{weight of individual solid waste}\times \text{emission factor},\left(\mathrm{kg}\;CO_{2}/\mathrm{kg}\right)$$

**Table 7.** Emission factor for different types of solid. ^21^

Types of waste	Weight, (kg)	Emission factors	Unit
Metal	7.77	3.23	kg CO _2_/kg
Water bottles	49.03	2.37	kg CO _2_/kg
Saline bottles	46.86	1.86	kg CO _2_/kg
Thick paper	72.12	1.12	kg CO _2_/kg
Thin paper	24.78	0.826	kg CO _2_/kg
Recyclable plastic	25.42	2.03	kg CO _2_/kg
Non-recyclable plastic	32.36	1.34	kg CO _2_/kg
Hard plastic	14.7	1.47	kg CO _2_/kg
Unbroken glass	24.07	0.868	kg CO _2_/kg
Others	38.61	1.68	kg CO _2_/kg
**Total non-risk waste generation/day**	**335.72**		
Infectious waste	118.72	3.68	kg CO _2_/kg
Gloves	44.44	1.34	kg CO _2_/kg
Tubing and bags	17.34	1.685	kg CO _2_/kg
Pathological waste	11.14	3.6	kg CO _2_/kg
Sanitary pads/Diaper	32.74	3.03	kg CO _2_/kg
Chemical waste	0.31	0.557	kg CO _2_/kg
Sharps (Glass)	11.16	0.868	kg CO _2_/kg
Sharps (Metal)	5.28	15.15	kg CO _2_/kg
Syringes	17.08	3.23	kg CO _2_/kg
**Total risk waste generation/day**	**258.21**		

For biodegradable waste,

Dhulikhel Hospital generates 167.78 kg of biodegradable waste per day. Estimation of Global warming potential, from IPCC 2006 guidelines (IPCC, 2006), default method (Tier 1) is used to determine the CH _4_ emission from landfill.

$${\mathrm{CH}}_{4}\;\text{emission}\;\left(\mathrm{Gg}/\mathrm{yr}\right)=\left[\left(\text{MSWT}\times \text{MSWF}\times \mathrm{Lo}\right)-R\right]\times \left(1-\mathrm{OX}\right)$$

where,

MSWT = Total MSW generated (Gg/yr) MSWF = Fraction of MSW disposed at SWDS

Lo = Methane generation potential = [MCF × DOC × DOCF × F × 16/12 (Gg CH4/Gg waste)] MCF = Methane correction factor (fraction)

DOC = Degradable organic carbon [fraction (Gg C/Gg MSW)] DOCF = Fraction DOC dissimilated

F = Fraction by volume of CH _4_ in landfill gas R = Recovered CH _4_

OX = Oxidation factor (fraction)

The direct carbon dioxide (CO _2_) emissions from the treatment processes and final disposal of organic waste are considered biogenic and are not included in the carbon footprint accounting (IPCC, 2006). This is because organic waste is made up of carbon that was recently taken from the atmosphere by the plants that the waste originated from. When the waste is treated or disposed of, the carbon in the waste is returned to the atmosphere in the form of CO _2_, which is then taken up by new plant growth in a continuous cycle of carbon uptake and release.

### Estimating emissions from liquid waste generation

A medium-sized system having three phases of treatment (Anaerobic Baffled Reactor, Horizontal Wetland, and Vertical Wetland) with two systems operating together. To conclude the wastewater treatment process, the system includes a sludge drying bed. The wastewater treatment plant in Dhulikhel Hospital treats 160 m ^3^/d of wastewater each day. See Table 8 for the emission from wastewater.

**Table 8.** Table for total emission from wastewater.

Bo (kg CH _4_/kg BOD)	MCFj	EFj (kg CH _4_/kg BOD)	BOD (kg/m ^3^)	Total water consumed (m ^3^/d)	Wastewater generation (m ^3^/d)	Wastewater treatment efficiency	Reference
0.6	0.8	0.48	0.22	200	160	70%	^22^

CH _4_ Emission factor for Wastewater Treatment/Discharge Pathway or System,

EFj=Bo×MCFj

where,

EFj = emission factor, kg CH _4_/kg BOD

j = each treatment/discharge pathway or system

Bo = maximum CH4 producing capacity, kg CH4/kg BOD = 0.6

MCFj = methane correction factor (fraction) = 0.8

$$\text{Total emission from waste water}\;\left(\mathrm{kg}\;{\mathrm{CH}}_{4}/\mathrm{BOD}\;d\right)=\text{Emission factor}\;\left(\mathrm{EFj}\;\left(\mathrm{kg}\;{\mathrm{CH}}_{4}/\mathrm{kg}\;\mathrm{BOD}\right)\right)\times \mathrm{BOD}\;\left(\mathrm{kg}/m^{3}\right)\times \text{Wastewater Generation}\left(m^{3}/d\right)\times \text{Wastewater treatment efficiency}$$

## Results

### Emissions generated by the use of generators

**Figure 2.**
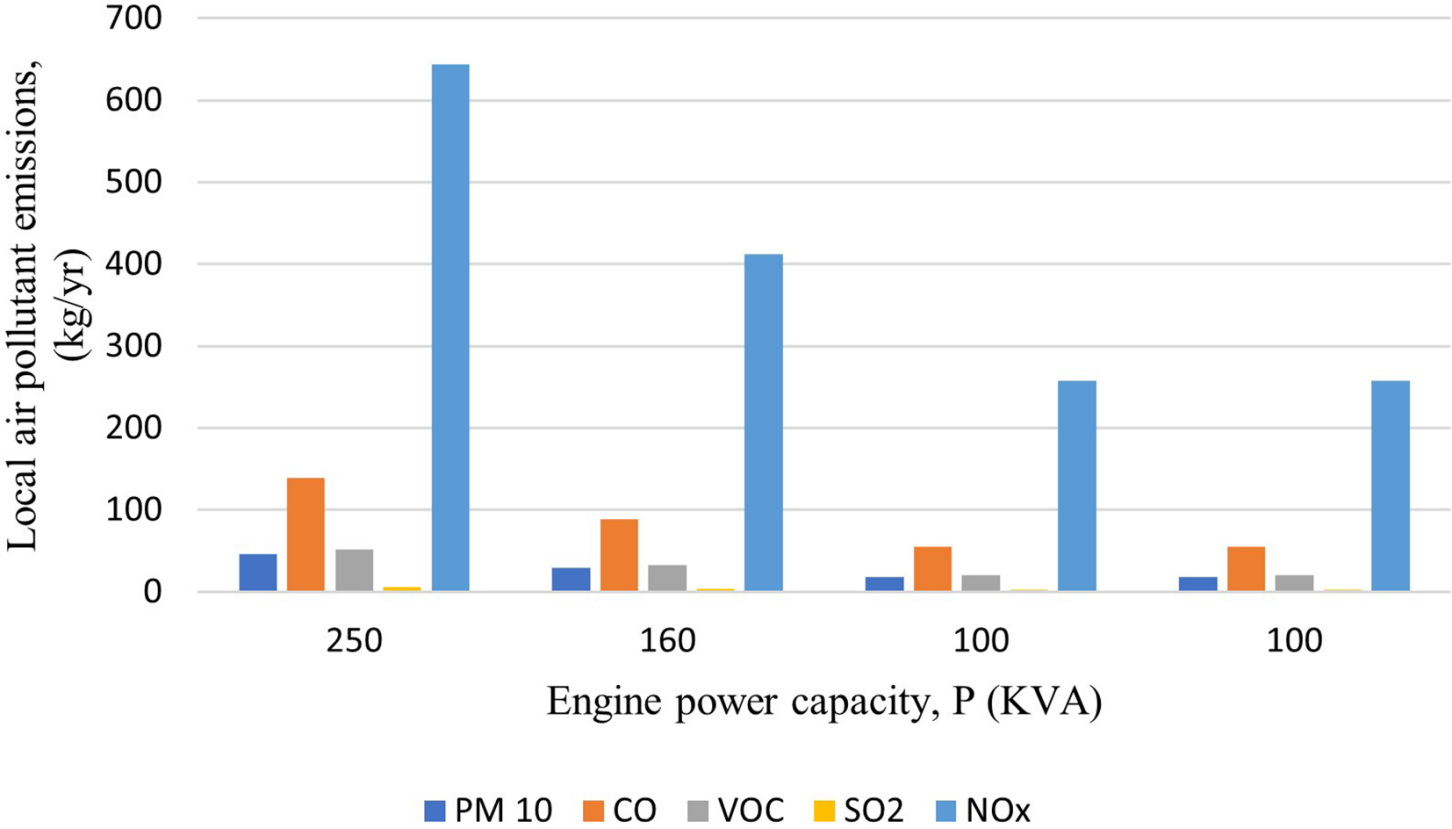
Emission of local pollutants from Generator.

**Figure 3.**
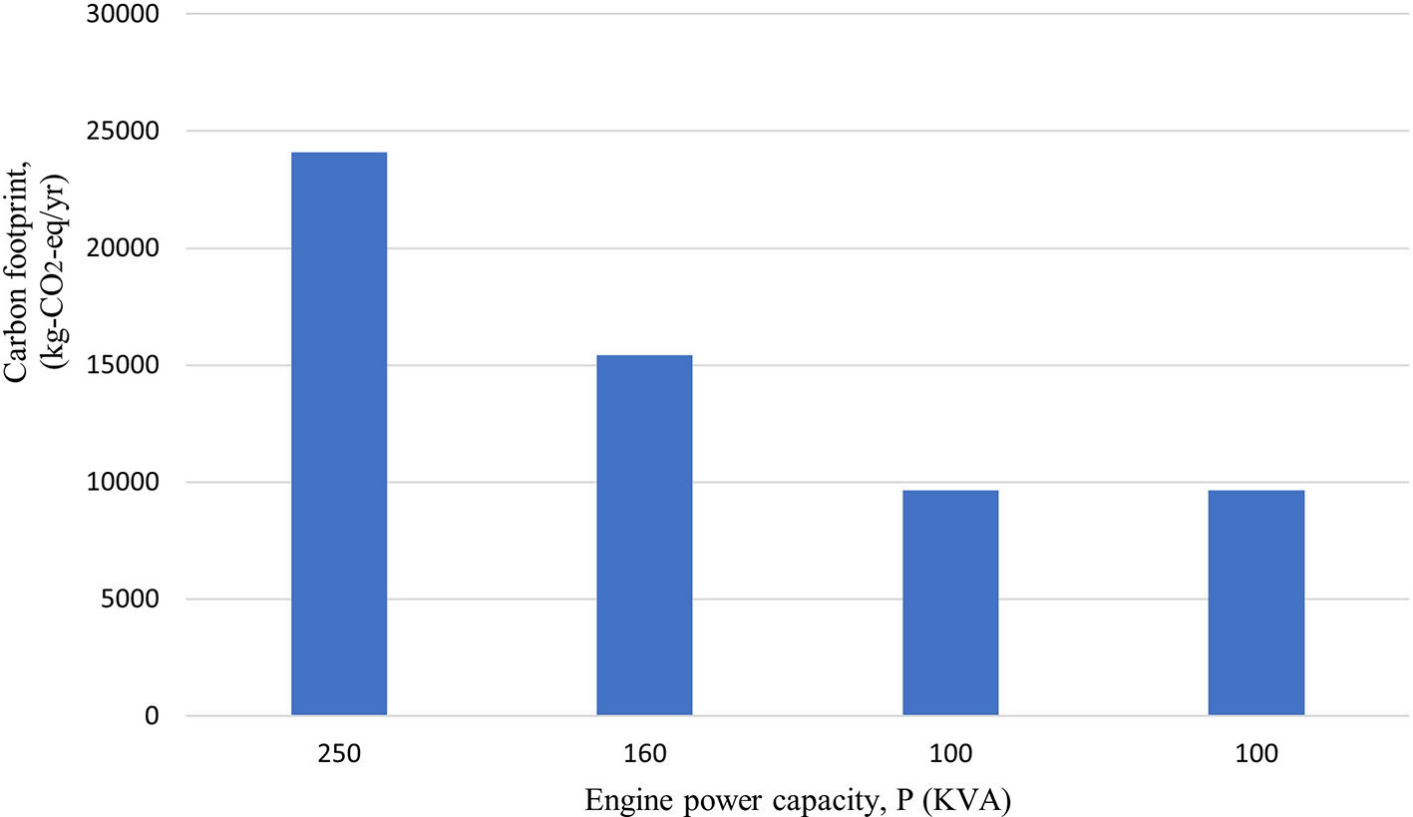
Carbon footprint from Generator.

Total carbon emissions from the generators were found to be 58,780 kg-CO _2_-eq/yr. Along with total CO _2_-eq, the total local air pollutants; PM _10_, CO, VOCs, SO _2_ and NO _X_ were found to be 112 kg/yr, 339 kg/yr, 125 kg/yr, 15 kg/yr and 1569 kg/yr, respectively. Figures 2 and 3 show the emission from the generators highlighting that the 250 kVA generator has the maximum emission. The emissions were found to be comparatively lower than the mentioned study as the generators is not operated in full time condition. The generators in hospital are operated in an average of 30 minutes per day (182.5 hours/yr) to ensure that they are working properly.

### Emission generated by the electricity consumption

**Figure 4.**
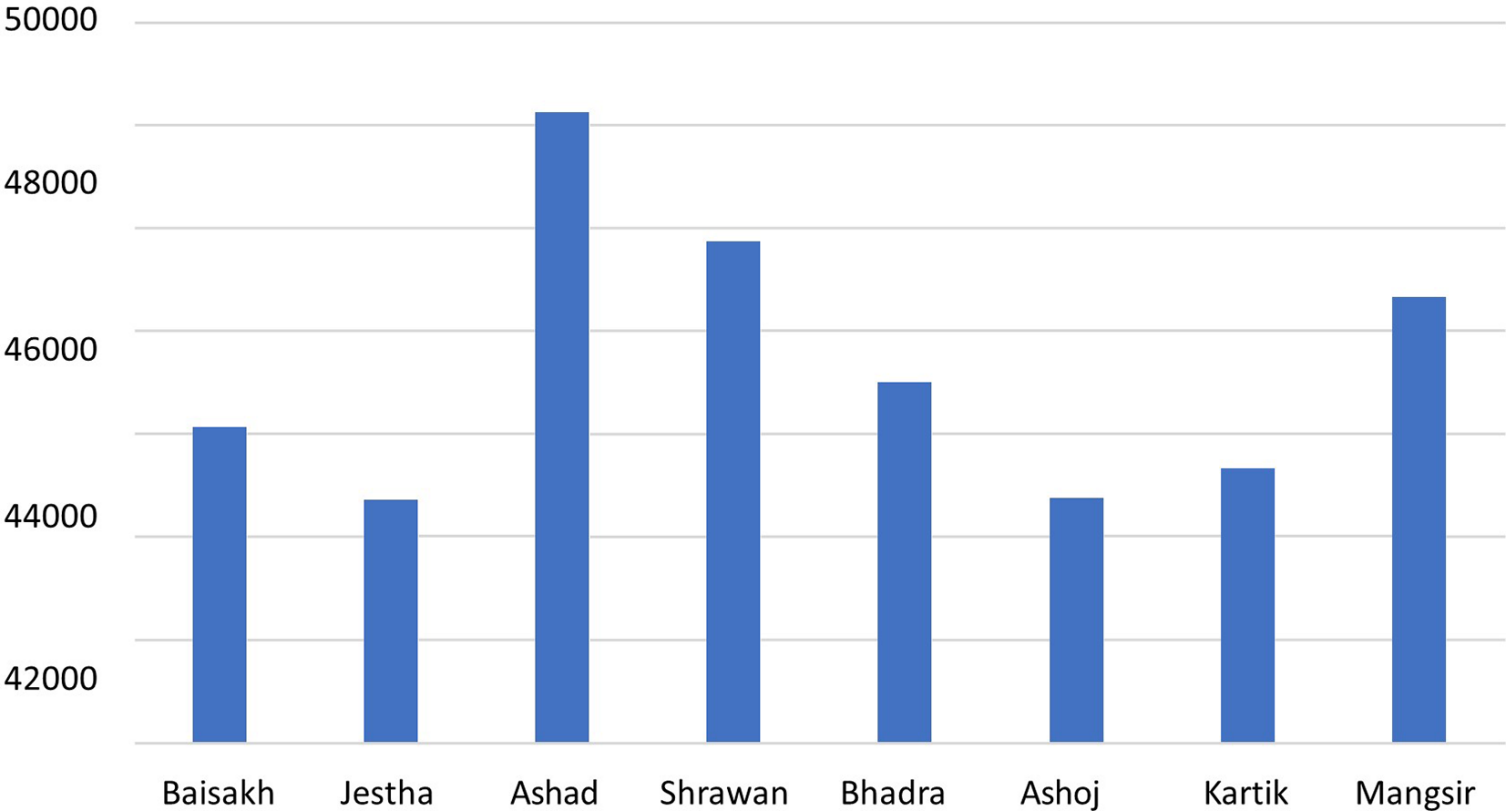
Carbon footprint from total energy consumption.

According to a study by the International Hydropower Association (IHA), the carbon footprint of hydropower is about 24 g-CO _2_-eq/kWh, which is much lower than the carbon emission of fossil fuels like coal (over 1000 g-CO _2_-eq/kWh). ^23^ The hospital annually consumes 1,942,845 KWh electricity, and with the imported electricity carbon footprint (see Data File ^27^), the GHG emission of electricity consumption was found to be 519,794 kg-CO _2_-eq/yr. The carbon emission is found to be highest in the month of June–July as shown in Figure 4, as the electricity consumption rate was highest.

The total carbon emission of Dhulikhel Hospital is quite high as its emission factor is the weighted average of electricity produced in Nepal and imports from India (coal), with T&D loss of Nepalese grid being 15%.

### Emission generated by the modes of transportation activities

**Figure 5.**
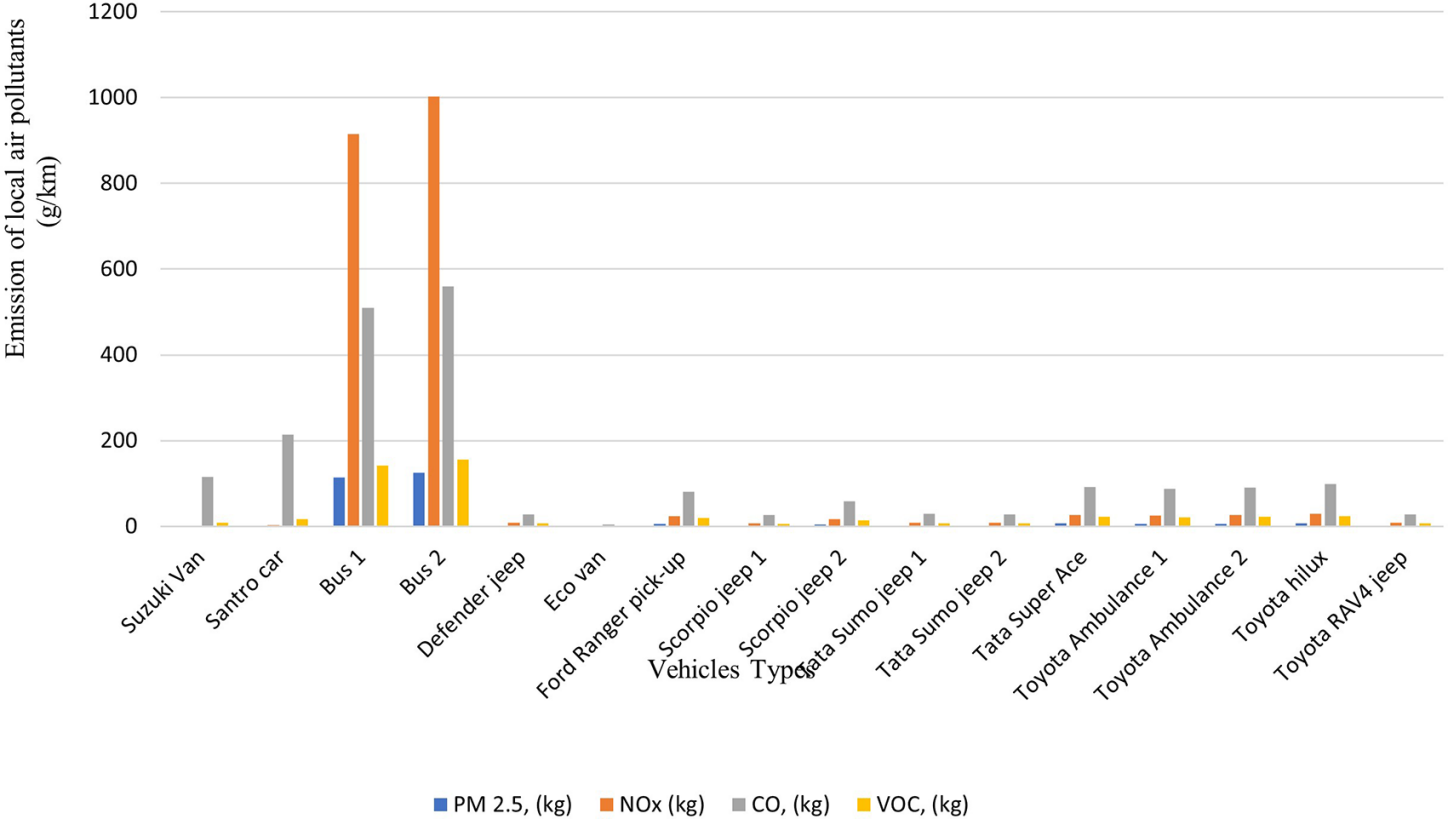
Emission of Local Air Pollutants from Transportation (in g/km).

**Figure 6.**
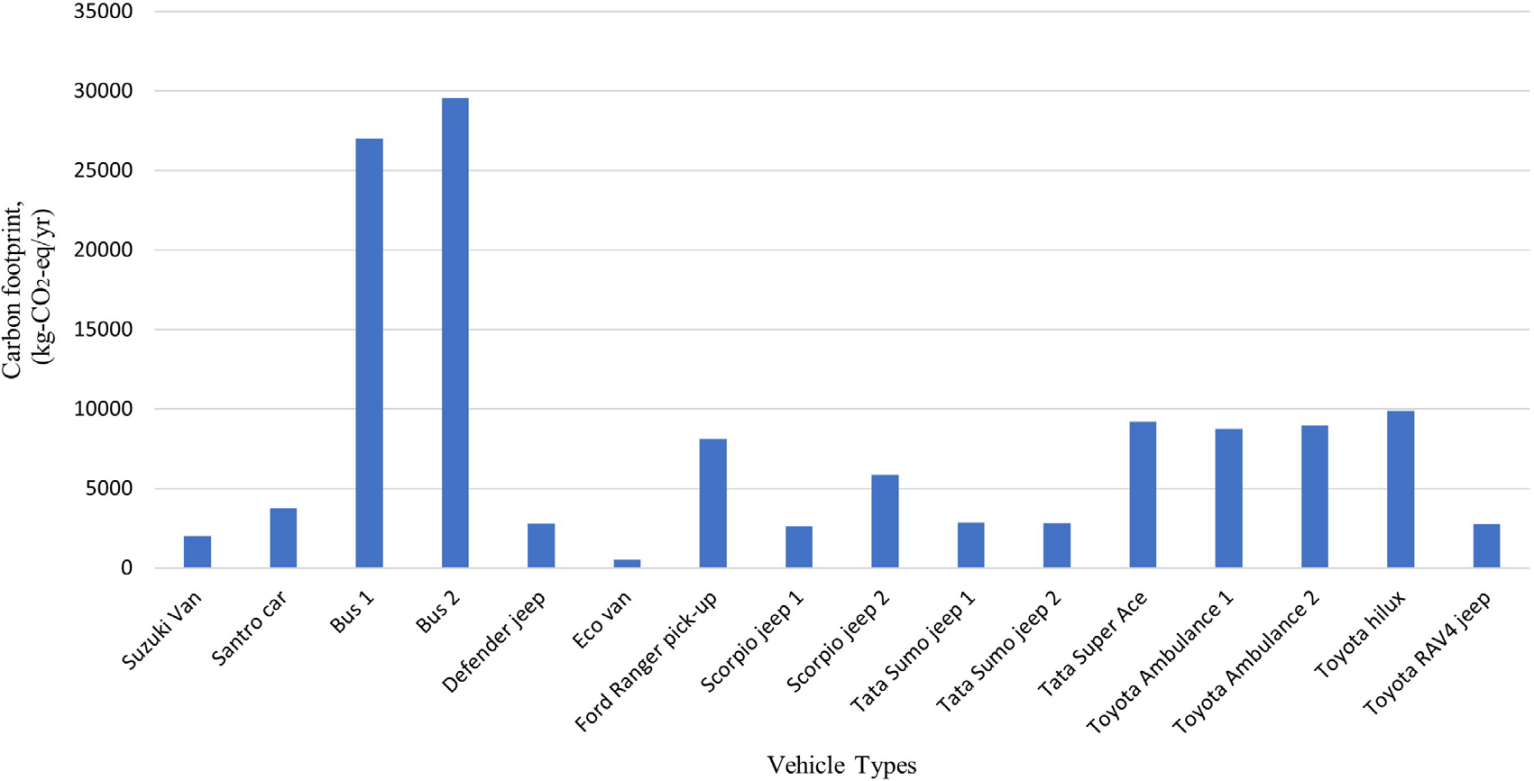
Emission of local Air pollution from Transportation (in kg).

The total carbon footprint of 16 hospital vehicles was found to be 127,453 kg-CO _2_-eq/yr, and the other local polluting agents: PM _2.5_, NOx, CO and VOC were calculated to be 289 kg/yr, 2115 kg/yr, 2053 kg/yr and 481 kg/yr, respectively. The buses used for staff transportation exhibit the highest levels of carbon emissions with 26,994 kg-CO _2_-eq and 29,582 kg-CO _2_-eq annually as shown in Figures 5 and 6. Compared to other vehicles used by the hospital, the buses also emit the highest levels of local air pollutants annually.

### Carbon footprint from transportation

**Figure 7.**
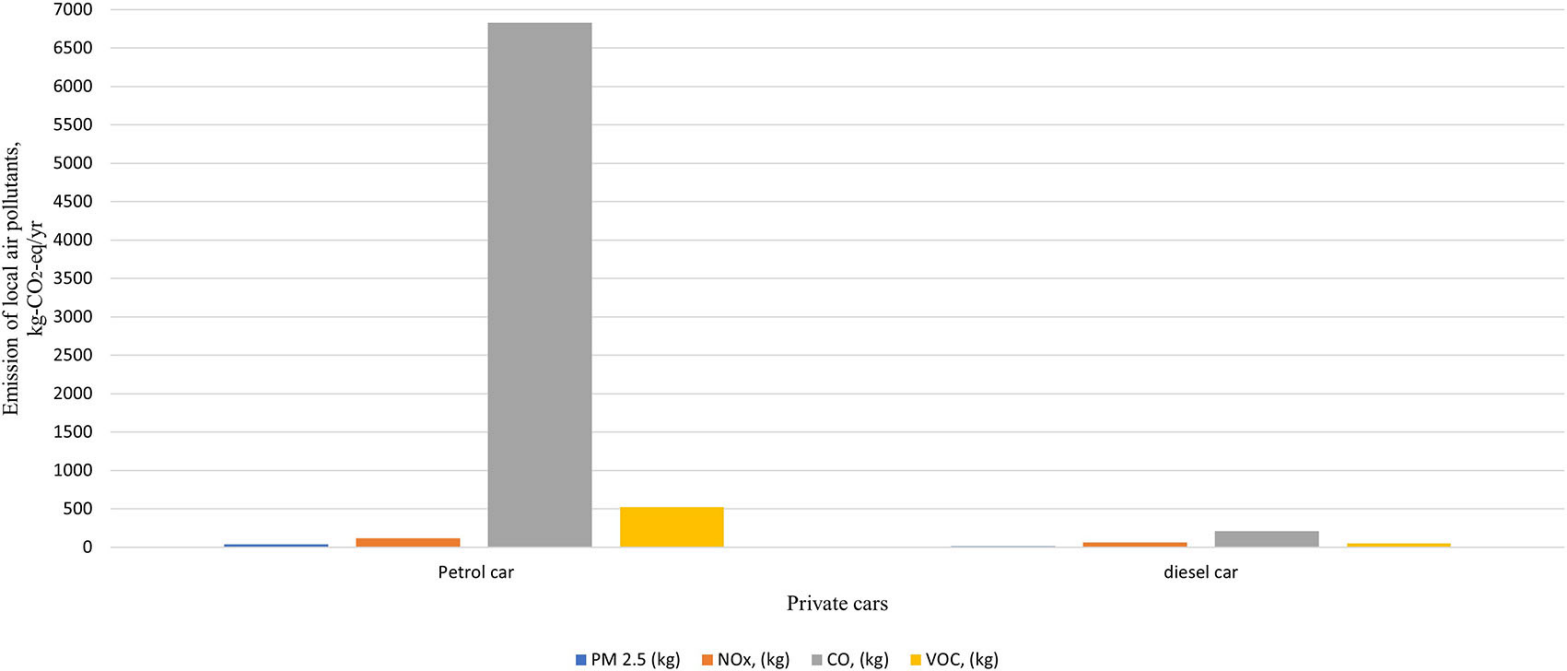
Emission of local air pollutants from private car.

**Figure 8.**
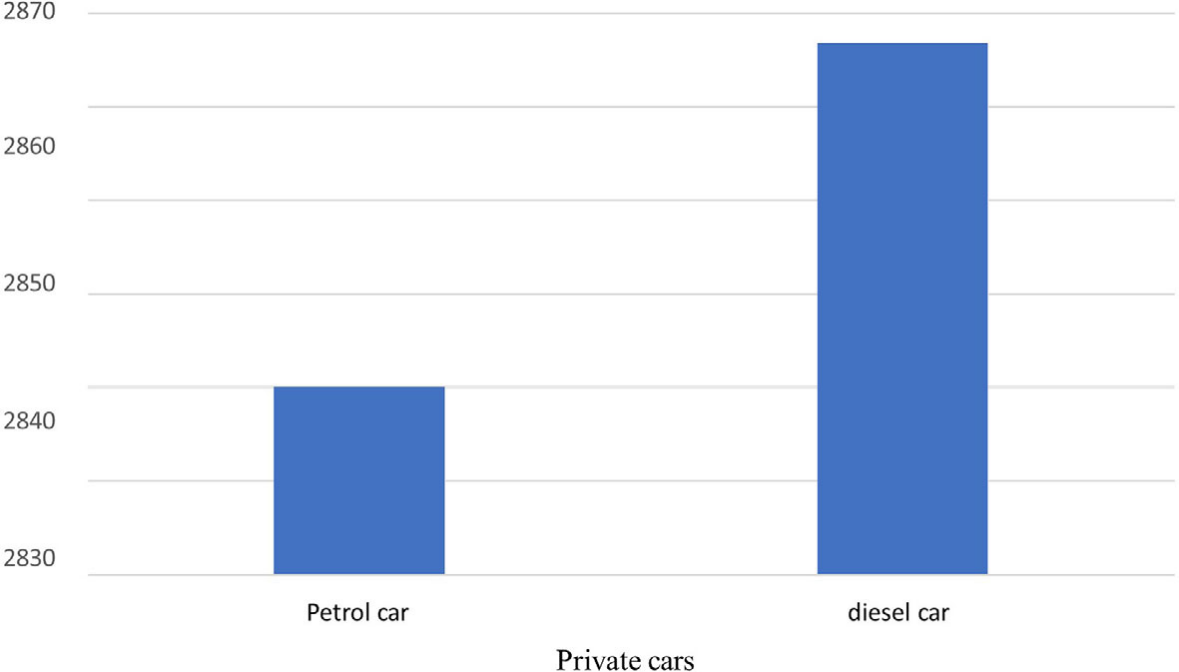
Carbon footprint from private cars.

The total carbon emissions from 44 Spark Ignition (SI) vehicles were found to be 124,523 kg-CO2-eq/yr, *i.e.,* one SI vehicle emits 2830 kg-CO2-eq/yr, while used for transportation of hospital activity only, while five Compression Ignition (Diesel) vehicles emit 14,334 kg-CO2-eq/yr, *i.e.,* one CI vehicle emits 2867 kg-CO2-eq/yr while used for transportation of hospital activity only. Diesel vehicles emit a slightly higher carbon footprint compared to Spark ignition vehicles due to their superior fuel efficiency. In comparison to other local polluting agents, the total amount of CO found was the highest, measuring 7039 kg/yr in total, as shown in Figures 7 and 8.

The carbon emissions from electric cars were found to be relatively lower, measuring 2022 kg- CO _2_-eq/yr, as compared to those from petrol and diesel cars because they do not have traditional internal combustion, they use battery as power source. So, electric vehicles (EVs) do not emit GHGs like CO _2_, CO, PM, NOx during their operation. EVs that release no greenhouse gases locally, are considered to be a potential response to the concerns of climate change and environmental pollution. ^24^

### Emission generated by the use of LPG cylinders

Liquified Petroleum Gas (LPG) is a clean alternative to traditional solid fuels that has received widespread attention due to its potential for scale. However, the climate, environmental, and health benefits or impacts of widespread LPG adoption in various low-income countries settings have not been thoroughly studied. ^25^ In our case, it can be observed that the total amount of LPG in 1020 cylinders consumed by hospital would be 15,912 kg. Then, using the carbon emission factor of 1.51 kg CO _2_ per kg of LPG burned, it is found that the carbon footprint from consumption of LPG would be 44494 kg CO _2_-eq/yr.

### Emission generated by the PV system

The hospital uses a PV system as a backup power source, operational during power cut-offs. Using the emission factor 123.8 g-CO _2_-eq/kWh, carbon emission was found to be 35,283 kg-CO _2_-eq/yr. Case studies like Bhagat Chandra Hospital and Gundersen Health System have successfully reduced their energy consumption by 20-30% and fully converted from fossil fuels to locally produced energy, resulting in annual operational savings of USD 3.7 million. ^26^ By incorporating 50 kW solar panels that are integrated with the electrical system, the hospital has reduced its energy consumption by 20–30% and avoided 93,000 kg of CO _2_ emissions since 2016. However, this option isn’t feasible in Nepal due to low carbon footprint of electricity from national grid in Nepal having low carbon intensity.

### Emission generated by the solid waste disposal

Based on an assessment of the waste composition at Dhulikhel Hospital, it has been determined that approximately 217.42 kilograms of waste per day may be suitable for autoclaving and subsequent reuse. However, certain types of hospital waste, including sharps such as needles, require additional processing such as shredding or melting to ensure their safe disposal. On the other hand, materials such as paper and plastic can be effectively recycled using anaerobic digestion and autoclaving, which has the potential to significantly reduce the overall emissions associated with waste management at the hospital. The hospital generates 167.78 kg of biodegradable waste per day, emitting 3822 kg CH _4_ per year. Organic waste from the hospital can be converted into energy through various techniques such as anaerobic digestion and composting. The energy recovery potential from anaerobic digestion of sorted and transported organic waste was calculated to be 2252 kWh.

**Figure 9.**
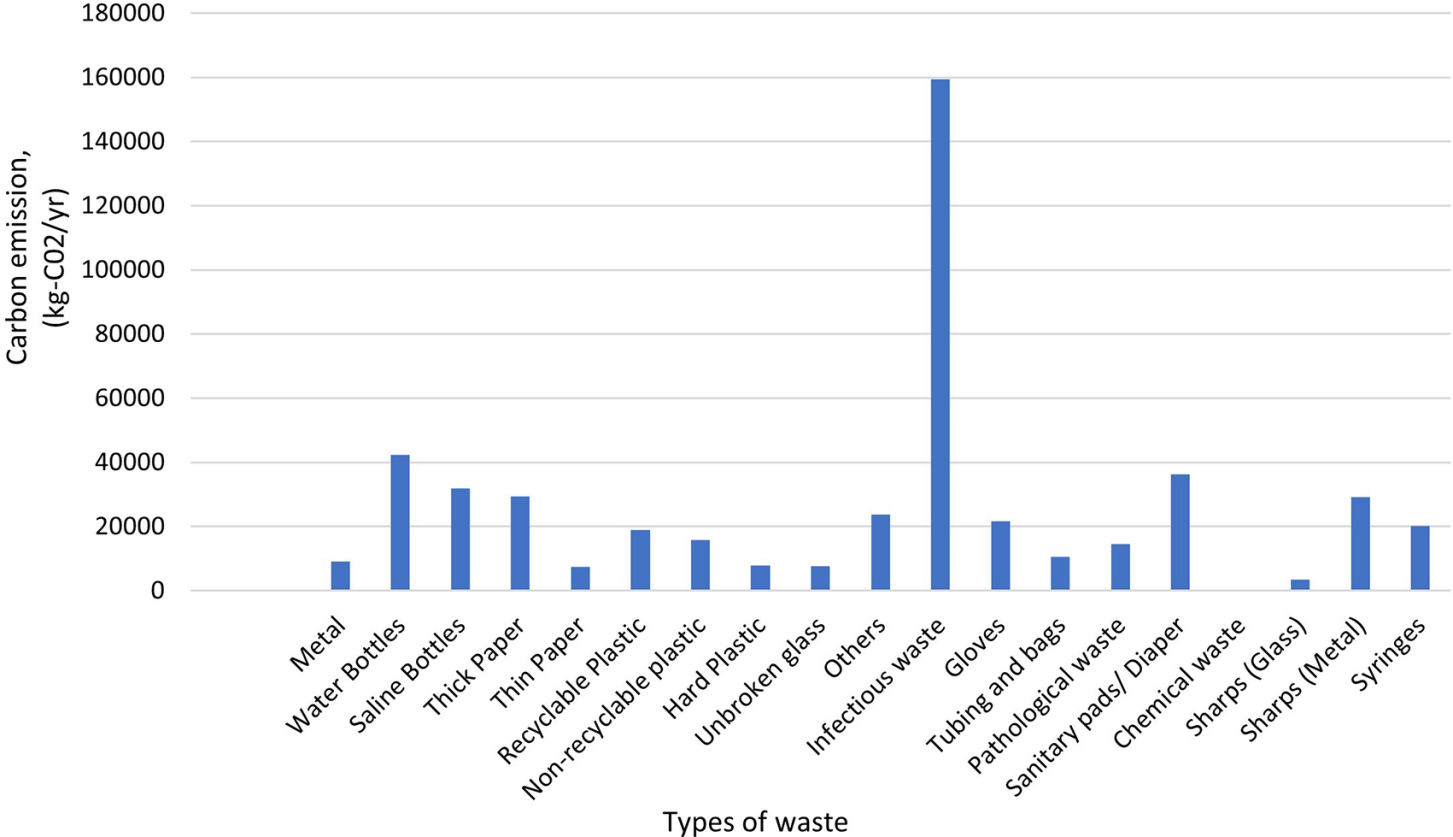
Carbon emission from solid waste.

According to the study, Dhulikhel Hospital generates an estimated 489,835 kilograms of carbon dioxide (CO _2_) annually through the disposal of its inorganic solid waste. Figure 9 depicts that hospital waste plays a significant role for greenhouse gas emissions, and highlights importance of implementing sustainable waste management practices to mitigate its environmental impact.

### Emission generated by liquid waste

Dhulikhel Hospital generates about 11.8272 Kg CH _4_/BOD liquid waste daily. Likewise, the emission factor of liquid waste has been determined to be 0.48 kg CH _4_/kg BOD. Based on these data, the total annual emission was estimated to be 4317 kg CH4/BOD yr for a wastewater treatment plant operating at 70% efficiency and generating 160 m ^3^/d of wastewater.

A gas collection system can be installed to capture and transport the methane gas produced during treatment to a biogas utilization system (a type of renewable energy system that converts organic waste into biogas, which can be used for heating, cooking, and electricity generation) to generate electricity or heat and minimize methane emission.

### Graphical representation of carbon footprint of different activities

**Figure 10.**
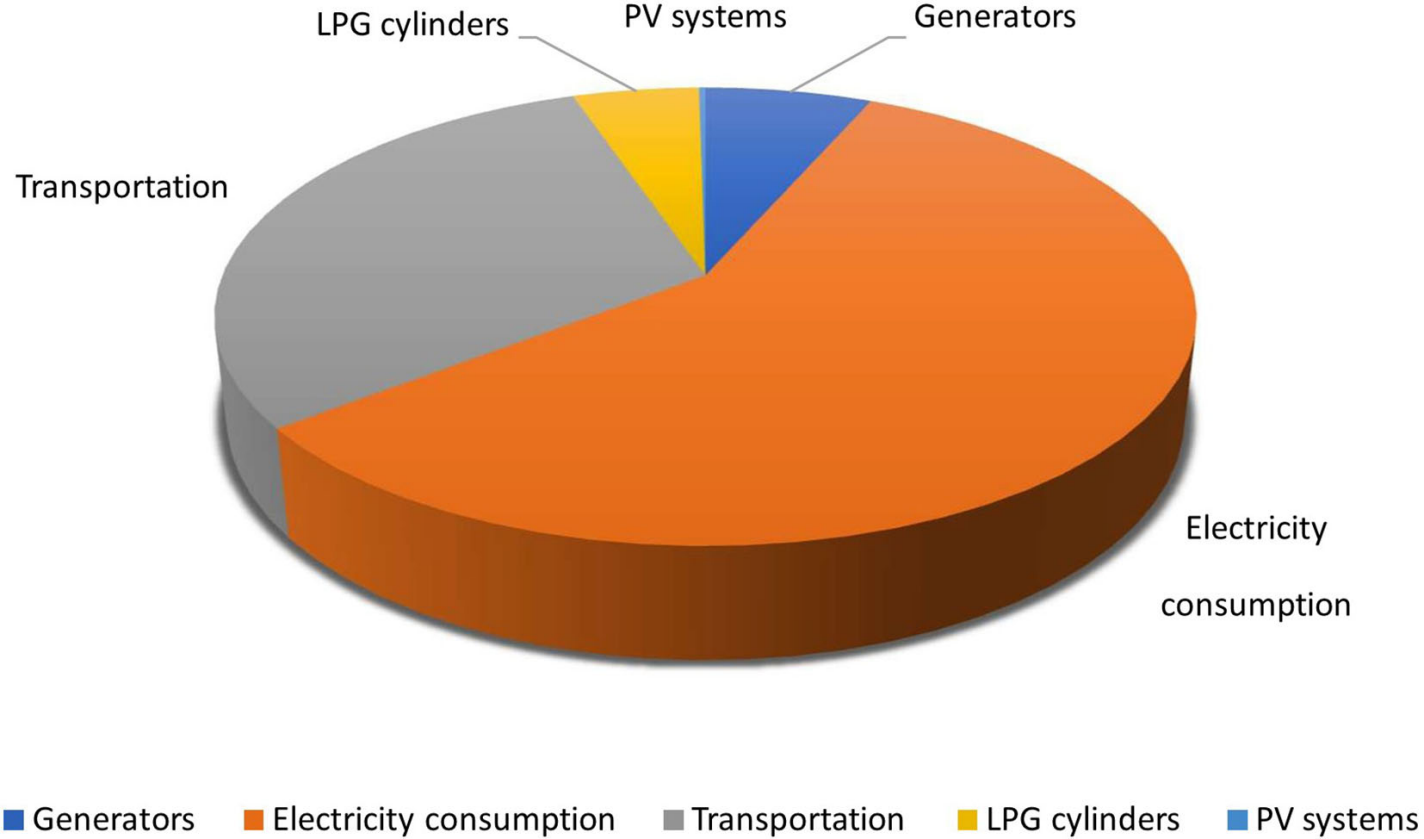
Carbon footprint from various activities throughout the year in the health care system.

According to the study, electricity consumption of the hospital was found to have high the solid waste produced by hospitals, which is primarily composed of inorganic materials, has been found to contribute significantly to greenhouse gas emissions, with an estimated total of 489,835 kg-CO _2_/yr. Electricity consumption has been found to have the highest carbon footprint among all the activities considered in the study as shown in Figure 10.

Additionally, the biodegradable waste generated by hospitals, such as food and organic matter, emits approximately 3822 kg-CH _4_/yr. Moreover, the liquid waste produced by hospitals, which contains significant amounts of organic matter, was found to emit 4316 kg-CH _4_/BOD/yr. These emissions of methane, a potent greenhouse gas, from hospital waste have a significant impact on the environment and contribute to climate change.

These findings highlight the importance of proper waste management practices in the healthcare industry, as reducing the amount of waste generated and effectively managing the waste that is produced can help mitigate the environmental impact of hospital operations.

## Discussion

### Existing energy scenario of Dhulikhel Hospital

From this study, it can be recapitulated that electricity consumption leads to CO2-eq emissions. The emission is accounted from the carbon intensity of national grid electricity of Nepal, which in turn uses carbon intensive electricity from Indian grid. Likewise, the environmental impact of transportation is more than that of photovoltaic system (PV system), Generator and liquefied petroleum gas (LPG) due to addition of use of conventional Spark Ignition (Petrol) and Compression Ignition (Diesel) vehicles for hospital transportation purposes.

### Benchmarking limits

This GtoG LCA study only performs static analysis and does not consider any dynamic variable changes, which may be crucial for future scenario analysis. However, no future scenarios have been studied in this study, a potential intervention for reducing emissions has been discussed. Furthermore, only GHG emission has been considered as an environmental impact category whereas freshwater eco-toxicity level (FETP), human-toxicity potential (HTP) and mineral depletion potential (MDP) could also be important. Similarly, gate-to-cradle (GtoC) LCA can incorporate recycling associated emissions as well as potential GHG avoid. This study sets as a foundation for further extending impact analysis.

### Comparison between real scenario and intervention

Dhulikhel Hospital has the potential to significantly reduce its carbon emissions by approximately 21,571 kg-CO _2_-eq/yr through the transition from diesel-powered buses to electric-powered ones. The terms “emission from the real scenario (A)” and “emission after intervention (B)” refer to two distinct measurements of emissions, as mentioned in Table 8. Specifically, “emission from the real scenario (A)” represents the actual emissions generated by staff transportation buses, while “emission after intervention (B)” represents the emissions resulting from the replacement of petrol and diesel buses with electric buses. The hospital's existing photovoltaic (PV) system can be used to substitute the conventional Nepal Electricity Authority (NEA) grid, which is powered primarily by electricity imports from India (coal sourced), for up to eight hours per day. The two sets of emissions data in Table 8 are labeled “Emission from real scenario (A)” and “Emission after Intervention.” The initial category relates to emissions produced when the PV system is not in operation, whereas the other refers to emissions measured after the PV system has been installed. This substitution can play a vital role in reducing the hospital's carbon footprint.

The actual scenario indicates that the maximum emissions are from transportation, electricity consumption, and solid waste generated by the hospital. After implementing interventions to reduce the amount of carbon dioxide and other greenhouse gases emitted into the atmosphere, the hospital can reduce its carbon footprint. See Table 9 for the emission generated in actual scenario and interventive scenario.

**Table 9.** Comparison between real scenario and Intervention.

	Emission from real scenario (A) (kg-CO _2_-eq/yr)	Emission after Intervention (B) (kg-CO _2_-eq/yr)	Emission reduction post- intervention (A-B) (kg-CO _2_-eq/yr)
For modes of transportation activities used for staff transportation	28,288.46	6717.78687	2,1570.67313
For the use of PV system for eight hours instead of NEA grid (supplied from india, coal)	444,173.4672	341,147.1072	103,026.36
Consumption of electricity from hydropower only	519,794.4614	51,923.196	467,871.2654

By relying solely on hydropower-generated electricity, the hospital could reduce its carbon footprint by an estimated 467871 kg-CO _2_-eq/yr. The two terms, “Emission from real scenario (A)” and “Emission after Intervention (B),” refer to two different measures of total emissions. Specifically, “Emission from real scenario (A)” denotes the total emissions resulting from energy consumption sourced from the NEA grid, which is supplied by coal from India. On the other hand, “Emission after Intervention (B)” represents the total emissions generated after energy consumption from hydropower, which is anticipated to be used in the near future. This reduction is primarily due to the fact that hydropower has substantially lower emissions in comparison to coal.

Intervention for reducing the carbon footprint in the waste sector of hospitals can involve implementing a “reduce, reuse, recycle, and compost” approach. This can be achieved by adopting a waste reduction and recycling program that involves segregating waste into different categories such as recyclable, organic, and hazardous waste. A system could be developed to ensure that each type of waste is managed appropriately.

By implementing this intervention, hospitals can significantly reduce their carbon footprint by diverting waste from landfills, which are major sources of greenhouse gas emissions. Recycling and composting can also reduce the need for new raw materials, which further reduces the carbon footprint of hospitals.

The study highlights the urgent need for the Nepalese healthcare system to adopt sustainable practices that reduce carbon emissions and mitigate their impact on the environment. The results of the study suggest that the Nepalese healthcare system, like many others around the world, is a significant contributor to climate change and its associated risks.

## Conclusions

Based on the findings of the study, several conclusions can be drawn. The total carbon emissions from the generators of the hospital were found to be 58,780 kg- CO2-eq/yr. In addition to the CO2-eq emissions, other local air pollutants were also measured, including PM _10_, CO, VOCs, SO2 and NO _x_. It is not possible to completely eliminate emissions generated by the operation of generators, as regular operation is necessary to maintain proper lubrication of its engine components and fuel circulation through the system. The hospital's electricity consumption from NEA grid was found to have a carbon footprint of 519,794 kg-CO _2_-eq/yr. The utilization of a PV system as a substitute for the conventional Nepal Electricity Authority (NEA) grid, which relies mainly on coal sourced from India, for a maximum of eight hours daily can have a significant impact on reducing the carbon footprint of the hospital. The combined carbon footprint of 52 private cars and 16 hospital vehicles was found to be estimated to be 272,374 kg-CO _2_-eq/yr. In addition to the carbon footprint, the study also measured the emission of other local pollutants such as PM _2.5_, NO _x_, CO, and VOC. Replacing only the petrol and diesel buses used for staff transportation with electric buses can result in a significant reduction in carbon footprint and also co-benefit local pollution. The hospital's use of LPG and PV system also contributed to the overall carbon footprint of the hospital. The study highlights the significant impact of Dhulikhel Hospital's inorganic waste on greenhouse gas emissions, emphasizing the need for sustainable waste management practices. Adopting appropriate waste management strategies that are suited to the specific waste stream characteristics, such as autoclaving and subsequent recycling or energy recovery, can greatly reduce the overall emissions associated with waste management. By implementing such practices, hospitals like Dhulikhel can contribute to mitigating the environmental impact of their operations and pave the way towards a more sustainable future. The hospital emitted 4317 kg CH _4_/BOD per year from its wastewater treatment plant. A gas collection system can be installed to capture and transport the methane gas produced during treatment to a biogas utilization system to generate electricity or heat and minimize methane emission.

## Data Availability

Zenodo: CARBON FOOTPRINT OF NEPALESE HEALTHCARE SYSTEM-A STUDY OF DHULIKHEL HOSPITAL. https://doi.org/10.5281/zenodo.8229778. ^27^ The project contains the following underlying data:
- Data File.xlsx Data are available under the terms of the Creative Commons Attribution 4.0 International license (CC-BY 4.0).
