# Supplementary material for: Carbon footprint of Nepalese healthcare system: A study of Dhulikhel Hospital
Source: F1000Res. 2024 Feb 5;12:1366. Originally published 2023 Oct 18. [Version 2] doi: 10.12688/f1000research.139552.2 (PMC10808856; doi:10.12688/f1000research.139552.2)
Supplement: Peer review history

# Reviewer report: Reviewer response for version 2

**DOI:** 10.5256/f1000research.162084.r243963

**Published:** 2024-02-21

**Authors:** Khem Gyanwali

**Affiliations:** 1 Department of Mechanical Engineering, Institute of Engineering, Tribhuvan University, GPO, Nepal

Accept the manuscript

Is the work clearly and accurately presented and does it cite the current literature?

Yes

If applicable, is the statistical analysis and its interpretation appropriate?

Yes

Are all the source data underlying the results available to ensure full reproducibility?

Yes

Is the study design appropriate and is the work technically sound?

Yes

Are the conclusions drawn adequately supported by the results?

Yes

Are sufficient details of methods and analysis provided to allow replication by others?

Yes

Reviewer Expertise:

Energy, Environment and Economics

I confirm that I have read this submission and believe that I have an appropriate level of expertise to confirm that it is of an acceptable scientific standard.

# Reviewer report: Reviewer response for version 1

**DOI:** 10.5256/f1000research.152834.r222215

**Published:** 2024-01-24

**Authors:** Sanjaynath Khanal

**Affiliations:** 1 Environmental Science, Madan Bhandari UNiversity of Science and Technology, Chitlang, Bagmati Province, Nepal

The article adequately puts the content and details of analysis and findings. However, I have the following observations for upgrading the article:

- Justify the study in Nepalese context with adequate background information.
- Method: Explain Functional unit/GtoG/mention about current and intervention analysis in methodology.
- Provide source of information on details of DH emission sources.
- Fig 1: Kathmandu not necessary. Mention specifics of collection, segregation and disposal.
- Fig 2: Should be in results.
- Table 9: Intervention analysis not mentioned above. Mention as appropriate.
- Fig 10: Does not include waste but mentioned in text. Update appropriately.
- Discuss why electricity consumption has the highest emission.
- Edit the text to make it more concise and focused.

Is the work clearly and accurately presented and does it cite the current literature?

Yes

If applicable, is the statistical analysis and its interpretation appropriate?

Yes

Are all the source data underlying the results available to ensure full reproducibility?

Yes

Is the study design appropriate and is the work technically sound?

Yes

Are the conclusions drawn adequately supported by the results?

Yes

Are sufficient details of methods and analysis provided to allow replication by others?

Yes

Reviewer Expertise:

Environmental Science

I confirm that I have read this submission and believe that I have an appropriate level of expertise to confirm that it is of an acceptable scientific standard.

# Reviewer report: Reviewer response for version 1

**DOI:** 10.5256/f1000research.152834.r219322

**Published:** 2024-01-23

**Authors:** Nawraj Bhattarai

**Affiliations:** 1 Department of Mechanical and Aerospace Engineering, IOE Pulchowk Campus, Tribhuvan University, Kathmandu, Nepal

1. The Authors have carried out Life Cycle Assessment LCA to determine carbon footprint from healthcare facilities. However, the type of LCA carried out is unclear. Is the LCA conducted a Static LCA or Dynamic LCA?
2. As the GtoG LCA of only one hospital has been carried out in this study, how do the findings generalize for Nepalese healthcare?
3.  It is mentioned that the emission reduction efficiency is zero and emissions are “Uncontrolled Emissions”. Clarification on what has been termed as “Uncontrolled Emission Values”.
4.  IC Vehicles have different fuel consumption on different load conditions/terrain situations. In the study single value has been used for fuel efficiency, why? 
5. As the Generators are used on average 30 minutes per day only to ensure proper functioning, has it been included in overall emissions due to having a significant footprint or considering that in case of power outage, its usage then would increase the overall carbon footprint of the Hospital?

Is the work clearly and accurately presented and does it cite the current literature?

Yes

If applicable, is the statistical analysis and its interpretation appropriate?

Yes

Are all the source data underlying the results available to ensure full reproducibility?

Yes

Is the study design appropriate and is the work technically sound?

Yes

Are the conclusions drawn adequately supported by the results?

Yes

Are sufficient details of methods and analysis provided to allow replication by others?

Yes

Reviewer Expertise:

Energy Policy, Environment, Renewable and Climate Change

I confirm that I have read this submission and believe that I have an appropriate level of expertise to confirm that it is of an acceptable scientific standard.

# Reviewer report: Reviewer response for version 1

**DOI:** 10.5256/f1000research.152834.r219318

**Published:** 2023-11-28

**Authors:** Kestutis Venslauskas

**Affiliations:** 1 Vytautas Magnus University, Kaunas, Lithuania

The paper has many serious limitations:

1. Abstract lacks the size of the hospital, there is no functional unit explained, no methodology given. Aim of study in abstract is wrong "... emissions from healthcare sectors" - you analyse only one small hospital, but not whole sector.
2. Text contains many typing errors, different measuring units, unidentified abbreviations (MRT, CT, CI), various indexes and subscripts errors.
3. Sentence "According to the study, recycling has the lowest carbon footprint per tonne of hospital waste (21–65 kg CO2-eq), followed by low temperature incineration with energy from waste (172–249 kg CO2-eq)." has wrong interpretation. If waste is incinerated, then it generates energy, and CO2 emissions should be negative.
4. Sentence "The primary objective of this study is to estimate the carbon print of hospitals" should be "footprint".
5. Electricity is not measured by KVA. kVA is power, but not electricity. As well "For its energy needs, the hospital relies on four solar panels, collectively possessing a capacity of 285 kWh" - PV capacity is measured by kWp of installed power, but surely not by kWh.
6. It is not clear how waste with Risk and no-Risk are separated or mixed together at collection stage and transportation?
7. Functional unit of LCA is not provided. LCA methodology is not clearly presented in internationally acceptable way.
8. Indicate emissions for "In the case of life cycle emission of solar systems, total capacity of the system was multiplied by the life cycle emission provided by Mehedi et al".
9. "Normalization of data was done according to the functional unit." - denote and describe your functional unit.
10. Fig 1 is completely wrong. a) diesel or petrol must go to Generator as well; b) arrow from Hospital should not go to Generator and PV system. Arrows must go from mentioned systems to Hospital. c) what is Generation (close to liquid waste)? d) why arrow goes to Operational consumables? e) Arrow must go from Supply from NEA to Electricity, but not wise-versa. f) Title of Fig 1 "Energy and material flow" - where is energy and materials? It is not indicated where is what.
11. "Additionally, data from medical research studies were analyzed to assess the environmental impact of certain treatments and procedures" - indicate what are "certain procedures".
12. Further from page 5, the methodology is not readable. It is not clear where are the explanations, where are the equations, there are no references for equations. Equations are wrong (e.g. The equation used for the estimation of the emissions - has  P = Engine power capacity rating (kW), but you indicate in kVA). Some text is repeated in several places the same. 
13. "Dhulikhel hospital has 16 different diesel and petrol vehicles for hospital services and 52 private cars in which 44 are found to be petrol cars, five are diesel and three are electric vehicles, found by random sampling" - how was it found by random sampling? Please give a table with parameters of vehicles.
14. Some tables: 2; 4; 5; 6; 8 can be explained in text by words and numbers.
15. In many cases not SI system units are used.

Is the work clearly and accurately presented and does it cite the current literature?

Partly

If applicable, is the statistical analysis and its interpretation appropriate?

No

Are all the source data underlying the results available to ensure full reproducibility?

Partly

Is the study design appropriate and is the work technically sound?

No

Are the conclusions drawn adequately supported by the results?

Partly

Are sufficient details of methods and analysis provided to allow replication by others?

No

Reviewer Expertise:

Environmental engineering, sustainability, LCA, renewable energy, bioenergy

I confirm that I have read this submission and believe that I have an appropriate level of expertise to state that I do not consider it to be of an acceptable scientific standard, for reasons outlined above.

# Reviewer report: Reviewer response for version 1

**DOI:** 10.5256/f1000research.152834.r222216

**Published:** 2023-11-16

**Authors:** Khem Gyanwali

**Affiliations:** 1 Department of Mechanical Engineering, Institute of Engineering, Tribhuvan University, GPO, Nepal

The present study holds contemporaneous and consequential significance within the domain of the Nepalese healthcare sector, presenting a prospective alignment with the governmental pursuit of achieving net-zero emissions. Nevertheless, several inquiries necessitate clarification. Primarily, the nature of the life cycle assessment (LCA) employed in this research warrants elucidation, specifically discerning whether it adopts a static or dynamic LCA methodology. The relevance of this distinction emerges notably in the evaluation and formulation of prospective scenarios, where a static LCA may lack meaningful applicability. It is imperative to ascertain the nature of the LCA utilized. Additionally, the importation of electricity from India by Nepal introduces a pertinent consideration. A comprehensive exploration of the authors' contemplation of this condition is warranted. Furthermore, an enhanced explication regarding the emissions stemming from vehicular sources is sought, with a particular focus on the potential ramifications in the event of alterations in material flow. Clarification on the envisaged trajectory of vehicular emissions amid shifts in material flow patterns is essential for a comprehensive understanding of the study's implications.

Is the work clearly and accurately presented and does it cite the current literature?

Yes

If applicable, is the statistical analysis and its interpretation appropriate?

Yes

Are all the source data underlying the results available to ensure full reproducibility?

Yes

Is the study design appropriate and is the work technically sound?

Yes

Are the conclusions drawn adequately supported by the results?

Yes

Are sufficient details of methods and analysis provided to allow replication by others?

Yes

Reviewer Expertise:

Energy, Environment and Economics

I confirm that I have read this submission and believe that I have an appropriate level of expertise to confirm that it is of an acceptable scientific standard, however I have significant reservations, as outlined above.

# Response

**Published:** 2023-11-16

**Authors:** Ashim Joshi

**Affiliations:** Kathmandu University, Dhulikhel, Central Development Region, Nepal

1) Though the LCA is not fully dynamic in nature the parameters considered for the analysis of future scenarios supports the dynamicity of the LCA within the considered boundary. 

 2)The electricity emission factor of Nepal used in the study is in consideration with the importation of electricity from India as well. 

 3) Definitely, the emission generated from vehicles changes as the material flow changes, especially during manufacturing phase. However, it was beyond the scope of the study to examine the future emission scenario of vehicles within the GtoG approach.
